# Effects of Different Cutting Styles on Physiological Properties in Fresh-Cut Carrots

**DOI:** 10.3390/plants13121665

**Published:** 2024-06-16

**Authors:** Ning Zhou, Sen Ma, Minwei Zhang, Jiayi Wang

**Affiliations:** National Demonstration Center for Experimental Biology Education, Xinjiang Key Laboratory of Biological Resources and Genetic Engineering, College of Life Science and Technology, Xinjiang University, Urumqi 830046, Chinazhang78089680@sina.com (M.Z.)

**Keywords:** metabolomic changes, cutting styles, antioxidant enzymes

## Abstract

With the internationalization of Chinese culture, ready-to-cook Chinese food has become popular. Vegetables in Chinese preparations are usually cut into slices, cubes, and shreds. Carrots, as a typical Chinese side dish, were selected as the model in this work. The polyphenol content, antioxidant capacity, O_2_^−^, hydrogen peroxide, malondialdehyde, lignin, antioxidant enzymes, and other enzymes activities were analyzed. The results indicated that these parameters were insignificantly different between three cutting styles. Therefore, metabolomics is further employed. Pathway enrichment indicated that glyceollin II and 6″-malonylgenistin were metabolites particularly expressed in the isoflavonoid biosynthesis pathway; (+)-gallocatechin, trans-chlorogenic acid, and (−)-epiafzelechin were specifically identified in the flavonoid biosynthesis pathway after slicing; and shredding caused the expression of coniferyl aldehyde and eugenol, which were specifically expressed in the phenylpropanoid biosynthesis pathway. These results indicate that different cutting styles do not change the physiological indicators of carrots but induce the expression of specific metabolites.

## 1. Introduction

Various fresh-cut products are easy to use and have become a new trend in fruit and vegetable consumption. As the internationalization of Chinese food advances, it has become popular worldwide. As a result, ready-to-cook foods are prepared. These products are packaged in packages of cut vegetables that consumers process in the kitchen without cutting or washing and are available in various cutting styles, including slices, cubes, and shreds.

The physiological metabolism of vegetables undergoes different changes depending on the cutting style. Hu et al. [1] investigated the effect of different injury intensities on the quality of fresh-cut pumpkin, and the results showed that brightness, whiteness index, respiration rate, ethylene content, lipoxygenase activity, and malondialdehyde content were increased while increasing the intensities, while the hardness, sensory quality, appearance, and total hardness were decreased. Shi et al. [2] investigated the effects of different cutting styles on the physicochemical properties and antioxidant activity of kiwifruit during storage at 4 °C for 4 days and found that the cutting styles had less effect on color, hardness, weight loss, total colony count, titratable acid, total suspended solids, and ascorbic acid content of kiwifruit compared to whole fruit. Guan et al. [3] explored the effect of cutting styles on the quality of fresh cucumbers cut into slices, cubes, and shreds, with whole cucumbers as the control. The results showed a gradual decrease in vitamin C content and a significant increase in glutathione content in sliced, cubed, and peeled cucumbers compared to those of whole cucumbers.

Carrots are rich in phenolics, sugar, fiber, and various vitamins [4]. They are used as a typical side dish in Chinese cuisine and in the preparation of dishes such as fish-flavored shredded pork and Kung Pao chicken. However, few studies have been conducted on the enzyme activity, antioxidant activity, and total phenolic content of freshly cut carrots using different cutting styles. This study aimed to explore the effect of cutting styles on carrots, with the results providing a practical basis for the industrialization of Chinese food.

## 2. Results and Discussion

### 2.1. Effect of Cutting Style on H_2_O_2_, O_2_^−^, and Malondialdehyde (MDA) Content of Fresh-Cut Carrots during Storage

**Figure 1:**
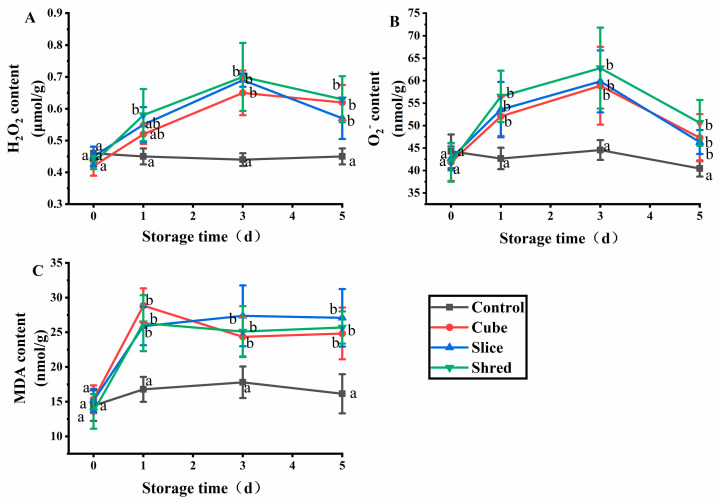
Effect of cutting styles on the (**A**) H_2_O_2_, (**B**) O_2_^−^, and (**C**) MDA contents of fresh-cut carrots during storage. Different lowercase letters in the same day indicate significant differences (*p* < 0.05).

When plants are damaged by biological and physical factors, receptors on the membrane receive and transmit stress signals to trigger oxidative bursts in plasma membranes and organelles and, finally, produce reactive oxygen species (ROS) [5]. ROS includes H_2_O_2_ and O_2_^−^ that can alter ion distribution and activate nuclear gene expression during signal transduction and amplification, thereby enabling plants to tolerate various stresses [6]. H_2_O_2_ and O_2_^−^ destroy macromolecules such as proteins, lipids, and nucleic acids, ultimately leading to cell death and the degradation of freshly cut fruits and vegetables [7]. After the three cutting treatments, the H_2_O_2_ and O_2_^−^ contents reached a maximum value (Figure 1B) at day 3 and then decreased in the subsequent days. As ROS are produced through various mechanisms, a key role is played by respiratory burst oxidase homologs (RBOHs). When plants are damaged by adversity, RBOHs are activated and produce a large amount of O_2_^−^, which is catalyzed by superoxide dismutase (SOD) to produce H_2_O_2_ [8]; therefore, when the carrot is cut, H_2_O_2_ and O_2_^−^ are preproduced in large quantities. When ROS accumulate to a certain extent, they have an impact on the normal physiology of plant cells; thus, cells initiate a series of nonenzymatic and enzymatic mechanisms to remove ROS [9]. The signals generated by the accumulation of ROS at a later stage promote the activity of SOD and catalase (CAT) to decrease the content of H_2_O_2_ and O_2_^−^. In the control group, the levels of SOD and CAT varied to a lesser extent and remained stable. On day 0, the content of H_2_O_2_ and O_2_^−^ in the control group was insignificantly different for the three cutting groups (*p* > 0.05). However, the H_2_O_2_ and O_2_^−^ content in the three cutting groups was significantly higher than those of the control group from the first day of storage (*p* < 0.05). However, no significant difference was observed between the different cutting styles (*p* > 0.05). The results showed that mechanical damage affected the production and metabolism of ROS in carrots, but different cutting styles had little effect on the production and metabolism of ROS.

MDA is the final product of membrane lipid peroxidation, and the MDA content reflects the degree of adverse damage suffered by plants [10]. In the three cutting groups, the MDA content was substantially increased from the first day of storage at 4 °C, indicating that a large number of superoxide radicals were generated when plants were subjected to adverse stress, leading to the peroxidation of membrane lipids and production of malondialdehyde (Figure 1C). Subsequently, MDA reacts with proteins and nucleic acids, rendering them nonfunctional and relaxing the bridge bonds between cellulose molecules or inhibiting protein synthesis, leading to cell membrane damage and membrane permeability changes [11]. After three days, the MDA content stabilized and was positively correlated with the H_2_O_2_ and O_2_^−^ content. The results showed that there was no significant difference (*p* > 0.05) in MDA content between the control group and the three cutting groups at day 0 (Figure 1C). However, the MDA content of the three cutting groups was significantly higher than that of the control from the first day of storage, but there was no significant difference (*p* > 0.05) among the three cutting methods.

### 2.2. Effect of Cutting Style on Antioxidant Enzymes and Antioxidant Capacity of Fresh-Cut Carrots during Storage

**Figure 2:**
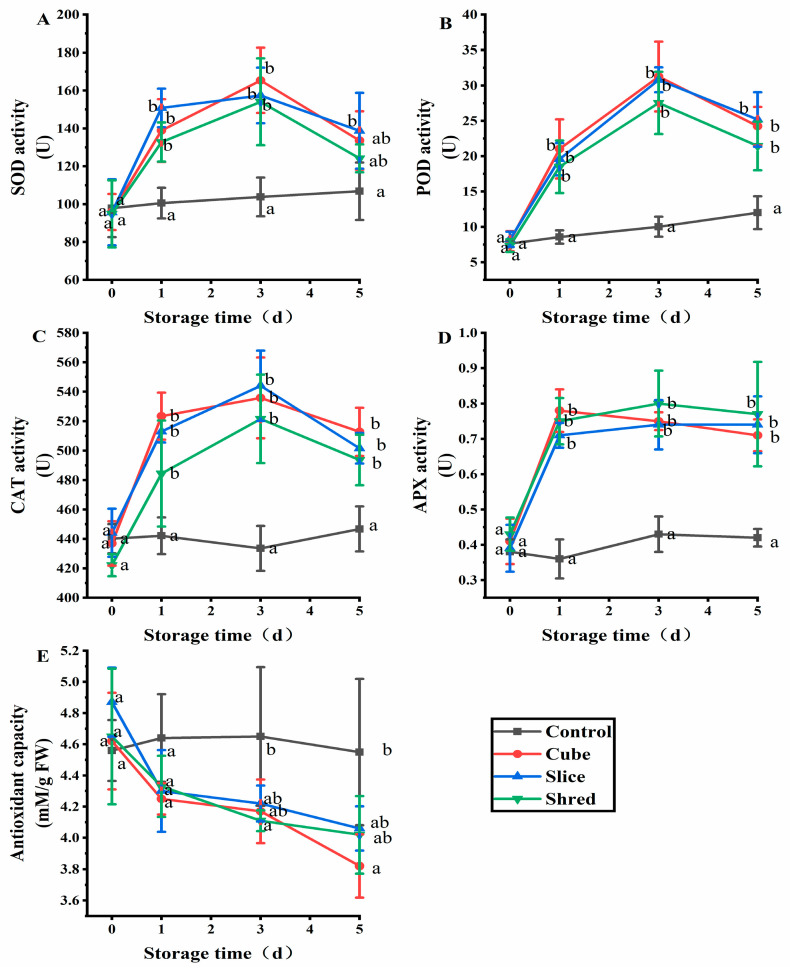
Effect of cutting style on (**A**) SOD, (**B**) POD, (**C**) CAT, and (**D**) APX activities, as well as (**E**) antioxidant capacity of fresh-cut carrots during storage. Different lowercase letters on the same day indicate significant differences (*p* < 0.05).

SOD is a naturally occurring superoxide radical scavenging factor that converts harmful superoxide radicals into H_2_O_2_ [12]. Afterward, H_2_O_2_ is decomposed into completely harmless water through the actions of CAT and POD [13]. In this way, the three enzymes together form a complete antioxidant chain [14]. SOD activity increased with the increasing O_2_^−^ content. The expression of these enzymes is promoted when mechanical injury occurs and the ROS content exceeds the physiological levels [15]. To keep the ROS content within the physiological range, SOD, peroxidase (POD), and CAT receive signals to increase their expression and activity and scavenge ROS. After the ROS content is reduced, the activities of these three enzymes gradually decreased; thus, their activities were positively correlated with the ROS content. After three cutting treatments, the three enzyme activities showed an increasing trend in the first three days of storage and were significantly higher than those of the control (Figure 2A–C). From day 3, the three enzymes showed a decreasing trend, but their levels were also significantly higher than those in the control.

APX, also known as vitamin C peroxidase, is an important antioxidant enzyme in plant cells that defends against external oxidative stress and regulates the metabolism of ROS. APX plays a crucial role in reducing oxidative damage caused by H_2_O_2_ [16]. APX has the strongest affinity for H_2_O_2_ [17]. The results showed that there was no significant difference (*p* > 0.05) in APX activity between the control group and the three cutting groups at day 0 (Figure 2D). However, the APX activity in the three cutting groups was significantly higher than the control from the first day of storage, but there was no significant difference (*p* > 0.05) among the three cutting groups.

The antioxidant capacity of the control group showed a balanced trend (Figure 2E), whereas the overall antioxidant capacity of DPPH in the three cutting groups showed a decreasing trend. On the first day of storage, the difference in antioxidant capacity between the control and the three cutting groups was not significantly different (*p* > 0.05). From the third day of storage, a significant difference was observed between the three cutting groups and control group (*p* < 0.05). Plant antioxidant capacity is used to scavenge ROS and MDA produced in plants, which leads to a decrease in antioxidant capacity.

### 2.3. Effect of Cutting Style on Phenylalanine Ammonia-Lyase (PAL), Polyphenoloxidase (PPO), Cinnamyl-Alcoholdehydrogenase (CAD), Lignin, and Total Phenol Content of Fresh-Cut Carrots during Storage

**Figure 3:**
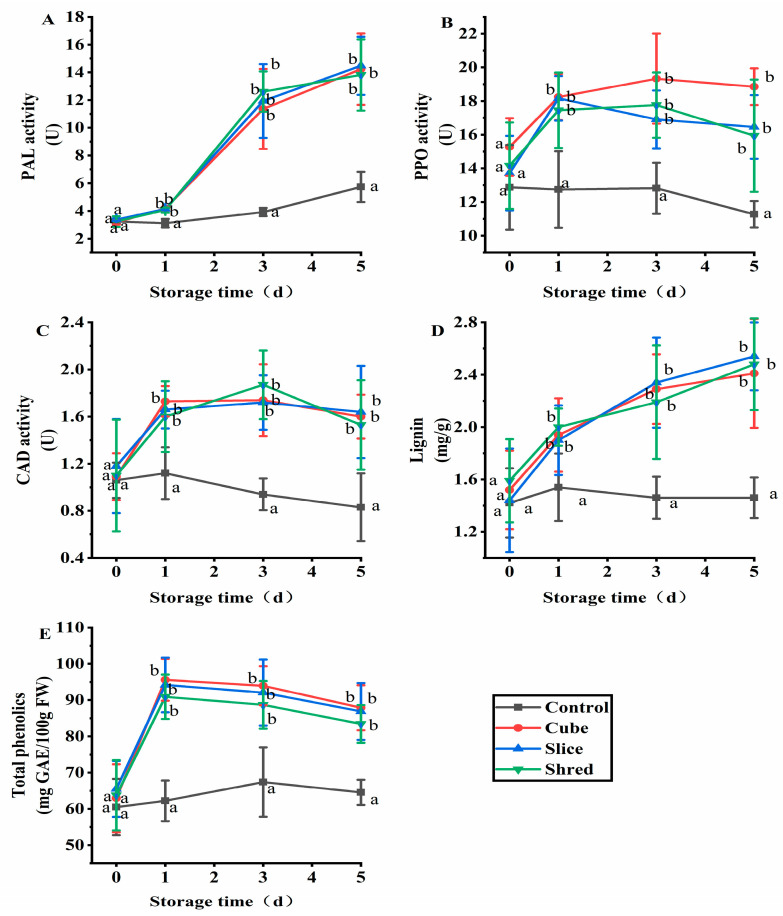
Effect of cutting style on (**A**) PAL, (**B**) PPO, and (**C**) CAD activities, as well as (**D**) lignin and (**E**) total phenolic contents of fresh-cut carrots during storage. Different lowercase letters on the same day indicate significant differences (*p* < 0.05).

Plants produce various secondary metabolites, including polyphenols, to adapt to adverse environments [18]. PAL is a key enzyme in phenylpropane metabolism, a biosynthetic pathway for many plant secondary metabolites, such as flavonoids, phenolics, lignin, and salicylic acid, which is closely related to plant resistance to stress and disease, being crucial in normal growth and development and in resisting pathogenic bacteria [19]. Increased PAL activity promotes the synthesis of polyphenolic substances in plants, resulting in an increased polyphenol content [20]. PAL enzyme activity increased throughout the whole storage period (Figure 3A). The polyphenol content increased significantly on the first day of storage and then slowly decreased on subsequent days (Figure 3E), which may be attributed to the enzymatic browning reaction using polyphenolic substances as substrates catalyzed by PPO (Figure 3B), where phenolics are converted into quinones [21]. PPO was recognized as tyrosinase, catecholase, and phenolase, which is widely distributed in nature, plant, fungal, and insect plastids [22]. After mechanical injury or pathogen contamination, PPO catalyzes the oxidation between phenols and O_2_, causing tissue browning [23]. PPO is related to food processing, storage, and the quality of fruits and vegetables, as well as tea quality and plant tissue culture [24].

Lignin is a phenolic polymer synthesized entirely by plants. Lignin is a primary component of plant cell walls, especially in vessel elements, xylem, hardened cells, thickened tissues, and the epidermis [25,26]. Lignin forms a complex network structure composed by three types aromatic compounds: H-lignin, G-lignin, and S-lignin [27]. These three types of lignin are derived from three core lignin monomers: p-coumaryl alcohol, coniferyl alcohol, and sinapyl alcohol, which are obtained through various enzymatic reactions. The precursors of lignin biosynthesis originate from phenylpropanoid metabolism [28]. PAL is the first enzyme involved in lignin synthesis [29]. PAL expression affects the entire lignin biosynthesis pathway, and its activity is positively correlated with lignin synthesis. In this work, both the lignin content (Figure 3D) and PAL activity (Figure 3A) showed an increasing trend. CAD is involved in the final step of lignin monomer synthesis, reducing the number of different cinnamaldehydes (such as sinap aldehyde, coumaric aldehyde, and coniferyl aldehydes) to the corresponding cinnamyl alcohols used to generate the precursors of lignin monomers. CAD is a key enzyme in the lignin synthesis pathway [30]. The trend of CAD activity in the three cutting groups increased and stabilized after day 1, being significantly higher than that in the control group (Figure 3C), consistent with the trend of the change in lignin content.

### 2.4. Metabolomic Changes

**Figure 4:**
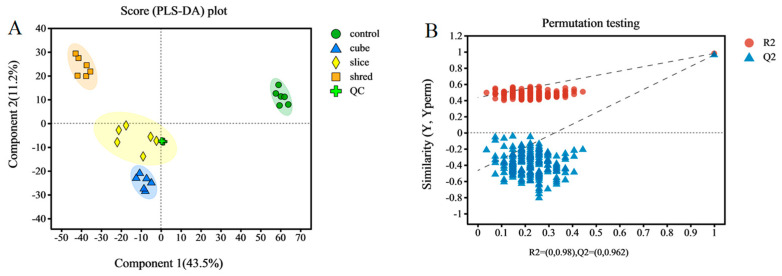
(**A**) Sample qualification analysis plots. PLS-DA score plots. (**B**) PLS-DA model validation.

From the above results of enzyme activity, antioxidant capacity, and other related indices, it can be concluded that there is no significant effect of different cutting styles on the quality of carrots. Therefore, a more in-depth metabolomic study was conducted to investigate the metabolic pathways and metabolite expression and accumulation in carrots that were specifically affected by different cutting styles. A good separation was obtained in the PLS-DA scores of the different treatment groups (Figure 4A), implying that the significant differences in metabolite species and levels between the different cuts were caused by the cutting styles. The cumulative interpretation rates of the PLS-DA model parameters were R^2^X = 0.651 and R^2^Y = 0.980, and the predictive ability of the model was Q^2^ = 0.879. This shows that the model is stable, reliable, and has a good predictive ability. To verify the reliability of the model, 200 iterative substitution tests were conducted. The intersection of the Q^2^ regression line with the *Y*-axis was on the negative semi-axis, further indicating that the PLS-DA model was stable and reliable (Figure 4B) [31].

As shown in Table 1, the common pathway of the three cutting styles was the phenylpropanoid biosynthesis pathway; however, the metabolites involved in the different cutting styles were different, and coniferyl aldehyde and eugenol were unique to the shredding style. In addition to the phenylpropanoid biosynthesis pathway, coniferyl aldehyde is involved in the metabolism and biosynthesis of secondary benzene metabolites. The isoflavonoid biosynthesis pathway was significantly enriched in the cubing treatment, and the metabolites involved were liquiritigenin, glyceollin II, and 6″-malonylgenistin. Liquiritigenin was also involved in the flavonoid and secondary metabolites biosynthesis pathways.

The flavonoid biosynthesis pathway was significantly enriched during the slice processing. There were five metabolites enriched to the pathway, namely (+)-gallocatechin, trans-chlorogenic acid, 5-p-coumaroylquinic acid, liquiritigenin, and (−)-epiafzelechin. These five metabolites are involved in the flavonoid and secondary metabolites biosynthesis pathways, while trans-chlorogenic acid and 5-p-coumaroylquinic acid are also involved in the phenylpropanoid biosynthesis, stilbene biosynthesis, diarylheptane, and gingerol biosynthesis pathways. The carotenoid biosynthesis pathway is a significantly enriched pathway shared by cubing and slicing styles. Four kinds of enriched metabolites were involved: (S)-abscisic acid, (+)-cis-abscisic aldehyde, abscisic acid, and dihydrophaseic acid. These four enriched metabolites were not found in the cutting style, and it was speculated that they were related to the enrichment of isoflavonoid and flavonoid biosynthesis in the cubing and slicing styles, respectively. In summary, in the significant enrichment pathway, the enriched metabolites unique to the cubing treatment were glyceollin II and 6″-malonylgenistin, whereas the specific enriched metabolites produced by slicing were (+)-gallocatechin, trans-chlorogenic acid, and (−)-epiafzelechin, while shred processing caused the enrichment of coniferyl aldehyde and eugenol metabolites.

## 3. Materials and Methods

### 3.1. Sample Processing

**Figure 5:**
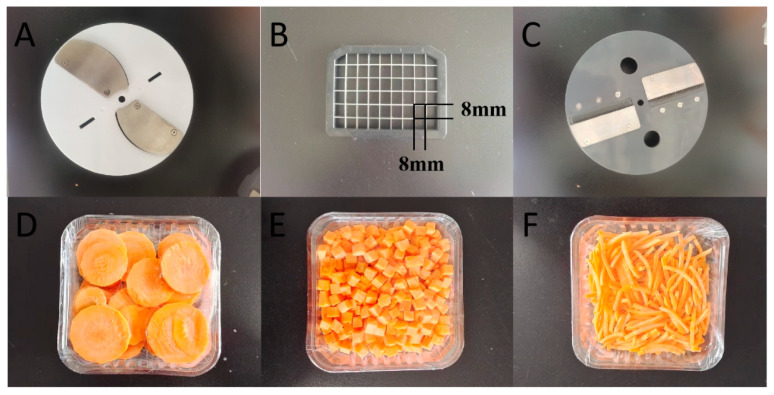
Schematic diagram of cube cutter blades. (**A**–**C**) are used for (**D**) slicing, (**E**) cubing, and (**F**) shredding, respectively.

Carrots purchased from the local market (Urumqi, China) were stored at 4 °C and used within five days. On the day of the experiment, samples with no mechanical damage, rotting, or deterioration and uniform size were selected, and the surface soil was cleaned with water. Samples were further peeled and cut into cubes, slices, and shreds using different modules (Figure 5) in a dicer (MDQ20; MUPOOL, Foshan, China). Intact, uncut carrots were used as a control. Samples were packed in 150 g preservation boxes (133 × 133 × 21 mm) and stored at 4 °C for five days. Samples were taken at 0, 1, 3, and 5 days and placed in an IKA liquid nitrogen grinder to prepare powders for further analysis.

### 3.2. Enzyme Activity Assay

The SOD levels were measured using a kit (G0101W; Gerace Bio, Suzhou, China). This kit employs the WST-8 method, which reacts with O_2_^−^ to generate a water-soluble formazan dye. This dye has a maximum absorption at 450 nm. SOD scavenges O_2_^−^ to inhibit the formation of formazan. The OD value of the reaction solution was negatively associated with SOD activity. When the percentage of inhibition in the xanthine oxidase coupling reaction system was 50%, the SOD enzyme activity in the reaction system was defined as one enzyme activity unit (U). 

The CAT levels were measured using a kit (Gerace Bio). CAT catalyzes the production of water and oxygen from H_2_O_2_, and the remaining H_2_O_2_ reacts with chromogenic probes to form colored products that have a maximum absorption peak at 510 nm. CAT activity in the sample was calculated by measuring the decrease in H_2_O_2_. The catalytic decomposition of 1 μmol of H_2_O_2_ per gram of sample per minute at 25 °C was defined as one enzyme activity unit (U). 

The POD levels were assayed using a commercial kit (Gerace Bio). Under the catalysis of POD, H_2_O_2_ oxidizes guaiacol to a red-brown product that has a maximum absorption of 470 nm. An increase of 1 absorbance value at 470 nm per gram of tissue per minute in the reaction was defined as one enzyme activity unit (U). 

The PPO levels were measured using a kit (Gerace Bio). PPO is a copper-containing oxidase that catalyzes the production of quinones, which have an absorption peak at 420 nm. An increase of 0.01 in the absorbance at 420 nm per gram of sample per minute in the reaction system corresponded to one enzyme activity unit (U).

The APX levels were measured using a kit (Gerace Bio). The oxidation of 1 μmol ascorbic acid per gram of tissue per minute at 25 °C was defined as one enzyme activity unit (U).

The PAL levels were measured using a commercial kit (Gerace Bio). This kit measures PAL activity based on the cleavage of L-phenylalanine into trans-cinnamic acid and ammonia. Trans-cinnamic acid has a maximum absorption value at 290 nm, and the rate of increase in absorbance is used to calculate PAL activity. An increase of 0.05 in the absorbance at 290 nm per gram of tissue per hour in the reaction system at 37 °C was defined as one enzyme activity unit (U).

The CAD levels were measured using a kit (Gerace Bio). CAD catalyzes the conversion of cinnamyl alcohol and nicotinamide adenine dinucleotide phosphate (NADP^+^) to cinnamaldehyde and triphosphopyridine nucleotide (NADPH), which then reacts with a specific chromogenic agent to produce a colored substance. The oxidation of 1 nmol of cinnamyl alcohol to 1 nmol cinnamaldehyde and the conversion of 1 nmol of NADP^+^ to 1 nmol of NADPH per milligram of histone per minute at 37 °C were defined as one enzyme activity unit (U).

### 3.3. Reactive Oxygen (ROS), Malondialdehyde, and Lignin Determination

Oxygen free radicals (OFR) were measured using a kit (Gerace Bio). O_2_^−^ reacts with hydroxylamine to produce NO^2−^ and NO^2−^ in the presence of p-aminobenzene sulfonic acid alpha-naphthylamine, producing a pink azo dye, which has a maximum light absorption at 540 nm. The content of O_2_^−^ in the samples could be calculated according to the value of A_540_, and the result was expressed as nmol/g fresh weight.

H_2_O_2_ was determined using a kit (Gerace Bio). H_2_O_2_ reacts with titanium salts to form a peroxide–titanium complex yellow precipitate, which can be dissolved using concentrated sulfuric acid and has a maximum absorption peak at 415 nm. The color shade was linearly related to the H_2_O_2_ concentration. The results were expressed as μM/g fresh weight.

The lignin levels were determined using a kit (Gerace Bio). Acetylation style was used to acetylate the phenolic hydroxyl group in lignin, which has a characteristic absorption peak at 280 nm. The absorbance value at 280 nm was positively correlated with the lignin content. The results were expressed as mg/g fresh weight.

The MDA levels were measured using a kit (Gerace Bio). MDA was condensed with thiobarbituric acid (TBA) under high temperature and acidic conditions to produce a red product with a maximum absorption peak at 532 nm, which could be used to estimate the content of lipid peroxide in the sample after colorimetry. The absorbance at 600 nm was measured, and the difference between the absorbance at 532 and 600 nm was used to calculate the MDA content. The results were expressed as nmol/g fresh weight.

### 3.4. Polyphenol and Antioxidant Activity Assay

The polyphenols levels were determined using the Folin–Ciocalteu method [32]. At room temperature and under light protection, Folin phenol reacted with polyphenol substances in the samples to produce a dark brown product with a characteristic absorption peak at 765 nm, which was positively correlated with the polyphenol content. The results were expressed as mg/100 g fresh weight.

The DPPH, FRAP, and ABTS assays are all chemical assays, and a similar trend was observed between these three methods in analyzing the antioxidant activity of fresh-cut produce [33,34]. Thus, previous studies [35,36,37] selected one method for analysis, and the DPPH method was used in this work to determine the antioxidant activity of fresh-cut carrot. The DPPH solution was dark purple in color, and its single electron captured the free radicals in the sample under room temperature and light protection, changing the color from dark purple to colorless after the reaction, with a characteristic absorption peak at 515 nm. The method reported by Wang et al. [32] was employed, and the results were expressed as μM/g fresh weight. 

### 3.5. Metabolomic Analysis

The sample at the end of storage (day 5) was used for metabolomic analysis. Liquid chromatography–mass spectrometry (LC-MS) was used to quantify carrot extracts using Thermo Fisher Scientific’s UHPLC-Q Exactive HF-X system with a HSS T3 column (100 mm × 2.1 mm, 1.8 µm). The data-dependent acquisition mode was used to collect the data. The detailed procedure for LC-MS was previously described by Gao et al. [38]. 

The raw LC/MS data were imported into Progenesis QI v.2.0 (Waters Corporation, Milford, CT, USA) software for peak alignment and peak picking. The KEGG and Majorbio Database were used as the primary databases for the search and identification of the metabolites. The R package ropls was used to perform PCA analysis. Additionally, Student’s *t*-test was performed. The *p*-value of Student’s *t*-test was used to select the distinctly different metabolites, and the metabolites with *p* < 0.05 were considered significantly different. Differential metabolites were annotated using the Kyoto Encyclopedia of Genes and Genomes database and mapped to Kyoto Encyclopedia of Genes and Genomes pathways.

### 3.6. Statistical Analysis

All experiments were biologically replicated three times. SPSS software v.20 (SPSS, Chicago, IL, USA) was used to analyze the experimental data univariately, and the significance test was carried out by the Duncan multiple comparison style. Differences were considered significant at *p* < 0.05. 

## 4. Conclusions

By analyzing the effects of different cutting styles on the physiological and metabolic indices of carrots, we found that the parameters of antioxidant enzymes, ROS metabolism, antioxidant activity, and lignin metabolism were insignificantly different between three cutting styles. At the metabolome level, the cube cutting causes changes in phenylpropanoid, isoflavonoid, and carotenoid biosynthesis, whereas slicing causes changes in phenylpropanoid, flavonoid, and carotenoid biosynthesis, and shredding can cause changes in phenylpropanoid biosynthesis. Among the significantly enriched pathways, the enriched metabolites unique to the cube style were glyceollin II and 6″-malonylgenistin, while those unique to the slice treatment were (+)-gallocatechin, trans-chlorogenic acid, and (−)-epiafzelechin. The shredding treatment resulted in the specific enrichment of coniferyl aldehyde and eugenol. In future studies, a cellular antioxidant assay should be used to more closely reflect the antioxidant capacity in vivo, as compared to the DPPH, FRAP, and ABTS methods. Moreover, the effects of preharvest factors on the physiological properties of fresh-cut carrots is an interesting topic and should be further determined in future studies.

## Figures and Tables

**Table 1:** Effect of the cutting method of fresh-cut carrots on the metabolite synthesis in selected metabolic pathways.

Treatment	KEGG Pathway Description	*p*-Value	Metabolite
Cube	Phenylpropanoid biosynthesis	0.04547	Cinnamaldehyde, Ferulic acid, Trans-Chlorogenic acid, 5-p-Coumaroylquinic acid, Coniferyl alcohol, P-Coumaraldehyde, Chavicol, 4-Vinylphenol, Coniferin, 1-O-Sinapoyl-beta-D-glucose
	Isoflavonoid biosynthesis	0.04768	Liquiritigenin *, Glyceollin II *, 6″-Malonylgenistin *
	Carotenoid biosynthesis	0.04768	(S)-Abscisic acid, (+)-cis-abscisic aldehyde, Abscisic Acid, Dihydrophaseic acid
Slice	Carotenoid biosynthesis	0.02913	(S)-Abscisic acid, (+)-cis-abscisic aldehyde, Abscisic Acid, Dihydrophaseic acid
	Flavonoid biosynthesis	0.03148	(+)-Gallocatechin *, Trans-Chlorogenic acid *, 5-p-Coumaroylquinic acid, Liquiritigenin, (−)-Epiafzelechin *
	Phenylpropanoid biosynthesis	0.04403	Cinnamaldehyde, Ferulic acid, Trans-Chlorogenic acid, 5-p-Coumaroylquinic acid, Coniferyl alcohol, P-Coumaraldehyde, Chavicol, 4-Vinylphenol, 5-Hydroxyferulic acid
Shred	Phenylpropanoid biosynthesis	0.005659	Cinnamaldehyde, Ferulic acid, Trans-Chlorogenic acid, 5-p-Coumaroylquinic acid, Coniferyl alcohol, P-Coumaraldehyde, Chavicol, Coniferin, 1-O-Sinapoyl-beta-D-glucose, 5-Hydroxyferulic acid, Coniferyl Aldehyde *, Eugenol *

* Metabolites specifically expressed in the corresponding pathways.

## Data Availability

Data are contained within the article.
